# Impact of foot-and-mouth disease on mastitis and culling on a large-scale dairy farm in Kenya

**DOI:** 10.1186/s13567-015-0173-4

**Published:** 2015-04-16

**Authors:** Nicholas A Lyons, Neal Alexander, Katharina DC Stӓrk, Thomas D Dulu, Jonathan Rushton, Paul EM Fine

**Affiliations:** The Pirbright Institute, Ash Road, Pirbright, Woking, GU240NF UK; Veterinary Epidemiology, Economics, and Public Health Group, The Royal Veterinary College, Hawkshead Lane, North Mymms, Hatfield, Hertfordshire, AL9 7TA UK; Department of Infectious Disease Epidemiology, MRC Tropical Epidemiology Group, London School of Hygiene and Tropical Medicine, Keppel Street, London, WC1E 7HT UK; State Department of Livestock, Ministry of Agriculture, Livestock and Fisheries, P.O. Private Bag Kabete, Kangemi, 00625 Nairobi Kenya

## Abstract

Foot and mouth disease (FMD) is a highly transmissible viral infection of cloven hooved animals associated with severe economic losses when introduced into FMD-free countries. Information on the impact of the disease in FMDV-endemic countries is poorly characterised yet essential for the prioritisation of scarce resources for disease control programmes. A FMD (virus serotype SAT2) outbreak on a large-scale dairy farm in Nakuru County, Kenya provided an opportunity to evaluate the impact of FMD on clinical mastitis and culling rate. A cohort approach followed animals over a 12-month period after the commencement of the outbreak. For culling, all animals were included; for mastitis, those over 18 months of age. FMD was recorded in 400/644 cattle over a 29-day period. During the follow-up period 76 animals were culled or died whilst in the over 18 month old cohort 63 developed clinical mastitis. Hazard ratios (HR) were generated using Cox regression accounting for non-proportional hazards by inclusion of time-varying effects. Univariable analysis showed FMD cases were culled sooner but there was no effect on clinical mastitis. After adjusting for possible confounders and inclusion of time-varying effects there was weak evidence to support an effect of FMD on culling (HR = 1.7, 95% confidence intervals [CI] 0.88-3.1, *P* = 0.12). For mastitis, there was stronger evidence of an increased rate in the first month after the onset of the outbreak (HR = 2.9, 95%CI 0.97-8.9, *P* = 0.057).

**Electronic supplementary material:**

The online version of this article (doi:10.1186/s13567-015-0173-4) contains supplementary material, which is available to authorized users.

## Introduction

Any disease among livestock creates inefficiency in a production system with negative economic impact to farmers. This impact can be divided into direct and indirect losses [1]. Direct losses are associated with an animal having a disease whose consequences may be immediately visible (e.g. death, abortion) or latent (e.g. reduced fertility). Indirect losses can be divided into additional costs, such as through the use of vaccines for disease prevention, or lost revenue which may occur if a farm is under quarantine, restricting access to local markets [1]. For many animal diseases an accurate estimation of disease impact is difficult due to a lack of available data and the variability of production systems employed around the world.

Foot and mouth disease (FMD) is a viral condition of ruminants characterised by initial pyrexia followed by the development of vesicles on the tongue, hard palate, coronary band and interdigital region. Lesions are also common on the teats in lactating cows and a sudden milk drop is typically seen [2]. Sudden death may also occur in young calves secondary to an acute myocarditis [3]. FMD virus is well known for being highly transmissible, made evident by widespread outbreaks when introduced to disease-free susceptible populations [4-6]; the virus is widely distributed throughout Africa, South America and Asia [7].

The annual global economic impact of FMD has recently been estimated at US$11 billion (90% range US$6.5-21 billion) in endemic settings and an additional minimum of US$1.5 billion has been ascribed to virus incursions into FMD-free countries [8]. The latter impact may be considerably greater than this, given that the 2001 outbreak in the UK has been estimated to have cost $US9 billion [9]. Moreover, the direct impact due to production loss in endemic areas is likely to be considerably underestimated as this is based on data from studies only considering losses through deaths or decreases in weight gain, milk production and draught power [10-13]. A study from Turkey also considered fertility and culling related losses, but was based on an economic model utilising evidence gained through a survey of expert opinion rather than empirical data [10,14]. A Kenyan field study of a SAT1 outbreak on four commercial dairy farms in 1999 did consider a broader range of direct and indirect impacts and estimated the total losses from all four farms to be around US$468 000 (range 15 000–225 000) [15]. This study was limited by only considering losses occurring during the outbreak period, analysing herd level losses retrospectively though a post-outbreak survey and not considering background levels of disease and culling. Poor characterisation of all these effects, and a lack of available data, precludes a more accurate estimate of economic impact in endemic areas. It is important that data from real outbreaks in the field are collected to gain a more accurate depiction of FMD impact and so inform resource allocation by governments and individual farmers. This is particularly necessary in endemic settings where many infectious diseases are present and competing for resources.

In developed countries, mastitis is frequently referred to as the most economically important disease in dairy herds [16] and is also reported as a major cause of morbidity among smallholders in Eastern and Southern Africa [17]. Farm profitability is reduced through decreased milk production, increase in milk discard, treatment costs, and associated culling. In animals affected with FMD, viral infection and replication within the udder may occur and teat lesions are likely to increase the risk of bacterial infection leading to clinical and subclinical mastitis [18]. Mastitis is one of many factors important in determining herd culling or replacement rate which have major implications for herd profitability [19,20].

Kenya has the largest population of dairy cattle in East Africa [21] and is endemic for four serotypes of FMD virus (A, O, SAT1, SAT2) [22]. Although smallholder dairies are estimated to supply over 70% of the marketable milk in Kenya [23], large-scale farms are still a significant part of the Kenyan dairy industry, tending to be more resistant to seasonal changes in milk production due to adoption of effective fodder storage technologies [24]. Despite the importance of mastitis and culling on dairy herd profitability, these parameters are poorly characterised among farms affected with FMD in endemic settings, and this may lead to underestimation of disease impact.

In August and September 2012, an outbreak of FMD occurred on a large-scale dairy farm in Nakuru County, Kenya [25]. The aim of the current study was to use data from this outbreak to estimate the influence of FMD on the risk of developing clinical mastitis and of subsequent culling, utilising survival analysis methods to provide objective evidence for these parameters in an endemic setting.

## Materials and methods

### Study area and population

The study area and population have been described in detail elsewhere [25]. In brief, the data were from a 1600 hectare mixed arable and large-scale commercial cattle dairy farm. Normal numbers of FMD susceptible livestock on the farm were approximately 600 cattle, 100 sheep and 300 goats. The farm had no pigs, and perimeter fencing ensured minimal contact with wildlife. Small ruminants were kept in separate paddocks a few kilometres from the cattle preventing direct contact between them. Cattle were all extensively grazed in 18 different groups based on age, weight, production and pregnancy status. As soon as possible after birth, calves were placed into individual hutches up to the age of around eight weeks. There were five separately grazed lactating cow groups including three with cows of lower parity. All groups were supervised 24 h a day by at least one stockman for purposes of security and to monitor animal health and oestrus events. All cattle were uniquely identified with a number visible on an ear tag placed shortly after birth.

The farm’s income was mainly through milk sales and selling in-calf or freshly calved heifers to other dairy farms. Cattle gave birth all year around and all breeding was through artificial insemination utilising sexed semen. No bulls were present on the farm, and any male calves were sold within a few days of birth. All data pertaining to health events, breeding, and farm exit were recorded using InterHerd software (InterAgri, School of Agriculture, Reading, UK).

### FMD outbreak

The outbreak of FMD on the study farm has been described in detail elsewhere [25]. In brief, serotype SAT2 was detected by antigen ELISA by the National FMD Laboratory in Embakasi, Kenya, which was subsequently confirmed by the World Reference Laboratory, Pirbright, UK. The index case was reported on the 31^st^ August 2012. FMD cases were defined by demonstrating hyperptyalism with at least one other clinical sign consistent with FMDV infection (decreased milk yield, decreased feed intake, oral lesions, interdigital lesions, pyrexia), although not all recorded cases received a physical examination by farm staff due to the large numbers affected. Daily recording of FMD cases was made by the livestock manager in consultation with individual group stockmen using lists of ear tag identification numbers for each group. Examination of approximately ten clinical cases by one of the authors (NL) revealed severe lesions on the dental pad, tongue, interdigital space and teats. This was consistent with observations by farm staff treating the affected animals. No clinical cases of FMD were seen among small ruminants.

The last recorded case in the outbreak had onset on the 28^th^ September 2012. The last recorded outbreak on the farm occurred in July 2004 although the sample submitted on that occasion to the Kenyan National FMD Laboratory failed to detect any viral antigen. Only five animals present during the current outbreak were on the farm in July 2004 but no detailed records were available from the earlier outbreak.

All cattle on the farm were vaccinated with the locally available quadrivalent vaccine (O, A, SAT1, SAT2) approximately every four months. The date of the last vaccination was 22nd May 2012. Sheep and goats were not vaccinated. Previous analysis found very limited or no vaccine effectiveness in preventing clinical disease [25].

### Study design

In this cohort study of disease impact, the primary risk factor under consideration was being a clinical case of FMD. The primary outcomes were whether a cow developed clinical mastitis (defined by having a swollen quarter or the presence of visible changes in the milk) or was culled (defined by removal from the herd due to any disease or death). For the latter, cows were also defined as culled if the recorded reason was low milk production. The study population was all cattle present on the farm at some point during the outbreak period (31^st^ August – 28^th^ September 2012). The date of the index case (31^st^ August 2012) was the date of entry into the study unless animals were born during the outbreak in which case the date of birth was used. All animals were followed until exit from the herd or the end of the study period (22^nd^ August 2013). The farm recorded the reasons for herd exit routinely. If an animal was sold for breeding or meat with no associated health reason for exit, it was censored at the date of herd exit. For the clinical mastitis analysis, the study population was restricted to animals over the age of 18 months at the start of the outbreak, as this is considered the age when clinical mastitis becomes a possibility. Animals exited the cohort at their first clinical episode of clinical mastitis and did not re-enter the cohort.

Potential confounders for the association between being a case of FMD and culling or developing clinical mastitis included age, parity, stage of lactation, breed, and a recorded history of another disease in the 12 months prior to the beginning of the outbreak. Several breeds and cross-breeds were present on the farm. Breed classification was based on the proportion of pedigree from indigenous breeds compared to non-indigenous exotic varieties. Binary variables were created for whether an animal had been recorded as affected with a disease in the previous 12 months. Although the farm vaccinates against FMD, previous analysis demonstrated limited or no effectiveness against clinical disease. The routine schedule means that number of vaccinations is co-linear with age so vaccination was not considered as a separate variable in the analysis. Age was categorised according to quintiles, and the number of days in milk was classified at the start of the outbreak as pre-lactation (i.e. never lactated), early-lactation (<0-100 days), mid-lactation (101–250 days), late-lactation (251+ days) and dry. Cows calving during the outbreak were included in the early lactation category (<0-100 days).

### Statistical analysis

Hazard ratios were generated through Cox proportional hazard regression models to estimate the effect of being a case of FMD on the primary outcomes. Variables were considered for inclusion in the multivariable model if they were associated with the risk factor (being a case of FMD) and outcome (culling or developing clinical mastitis). Associations between variables and being a case of FMD were assessed through chi-square tests whilst association with subsequent culling and developing clinical mastitis was through calculation of rate ratios and likelihood ratio tests. Variables associated with the risk factor and outcome with *P*-values of <0.1 were retained for multivariable model building using a backward fitting approach. Age was included in all models as an a priori confounder. Prior to model building, likelihood ratio tests were used to assess for linear trends of risk factor variables where appropriate. Likelihood ratio test *P*-values of ≤ 0.05 were used to indicate sufficient evidence for the inclusion of the variable in the model. The proportional hazards assumption was assessed through examination of a combination of Nelson-Aelen plots and global Schoenfeld residual tests. Where evidence for non-proportional hazards was observed, Schoenfeld residuals for each indicator variable were generated alongside scaled Schoenfeld residuals plots to explore the non-proportionality. Based on these observations, variables were incorporated as time-varying effects with a choice of multiplier function based on the observed plots and Akaike information criterion (AIC) values. Repeated examination of Schoenfeld residuals and scaled residual plots were conducted to ensure the time varying effect was accounted for in the model incorporating the time-varying effect. Interactions between terms in the final model were tested through likelihood ratio tests.

All data were extracted from Interherd through Microsoft Access and imported into Stata 13.0 (Statacorp, Texas, USA) for analysis.

## Results

A total of 644 cattle were present at some point during the outbreak, including 26 born during this period, of which one was male. Four hundred and nine animals were at least 18 months of age at the start of the outbreak period and hence included in the mastitis analysis. Of all cattle present during the outbreak, 400 (62.1%) were recorded as clinical cases of FMD. Total follow-up time for mastitis was 3683.0 cattle-months (median 11.7, range 0.36-11.7 per animal) whereas for culling it was 6669.6 (median 11.7, range 0.16-11.7). During the follow-up period, 63 cattle developed clinical mastitis (incidence rate 17.1 per 1000 cattle-months, 95%CI 13.4-21.9). The total number of animals exiting the herd during the follow up period was 166, of which 76 left the herd due to disease, death or low production. The most common reason for culling was infertility (Table 1). The overall incidence rate for culling was 11.4 per 1000 cattle months (95% CI 9.1-14.3).

**Table 1:** **Reasons for exit and culling after a foot-and-mouth di** **se** **ase outbreak**

**Exit category**	**n**	**Column %**	ᅟ		
Exit herd	166	25.8	ᅟ		
Not exit herd	478	74.2	ᅟ		
Total	644	ᅟ			
**Reasons for exiting herd (** ***n*** **= 166)**					
Culling	54	32.5	**Reasons for culling**	**n**	**Column %**
			Infertility	31	57.4
			Mastitis	5	9.3
			Lameness	5	9.3
			Tick-borne disease	2	3.7
			Mastitis and fertility	2	3.7
			Poor condition	4	7.4
			Low production	2	3.7
			Other illness	3	5.6
Death	22	13.3			
Old age	2	1.2			
Behavioural	1	0.6			
Sold for meat	5	3.0			
Sold as breeding stock	82	49.4			
Total	166				

Dairy farm is located in Nakuru County, Kenya. Follow-up period is 12 months following the beginning of the foot-and-mouth disease outbreak. Animals are included if present on the farm at some point during the outbreak period (31^st^ August-28^th^ September). Culling is defined as exiting the herd due to any disease, death or low production.

**Figure 1:**
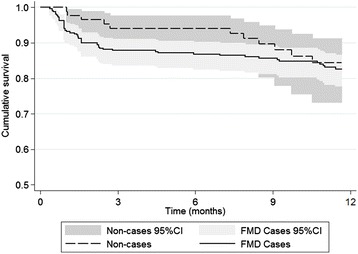
**Unadjusted Kaplan-Meier survival curve for FMD cases and non-cases related to developing clinical mastitis.** Dairy farm was located in Nakuru County, Kenya. Cattle were included in the analysis if present on the farm during the outbreak period (31^st^ August-28^th^ September 2012) and were followed for 12 months after the commencement of the outbreak. Cattle were included if over the age of 18 months at the start of outbreak, considered as the age when the outcome becomes a possibility. Log-rank test for equality of survivor function, *P* = 0.43. The Y-axis represents the cumulative probability of not having clinical mastitis.

**Figure 2:**
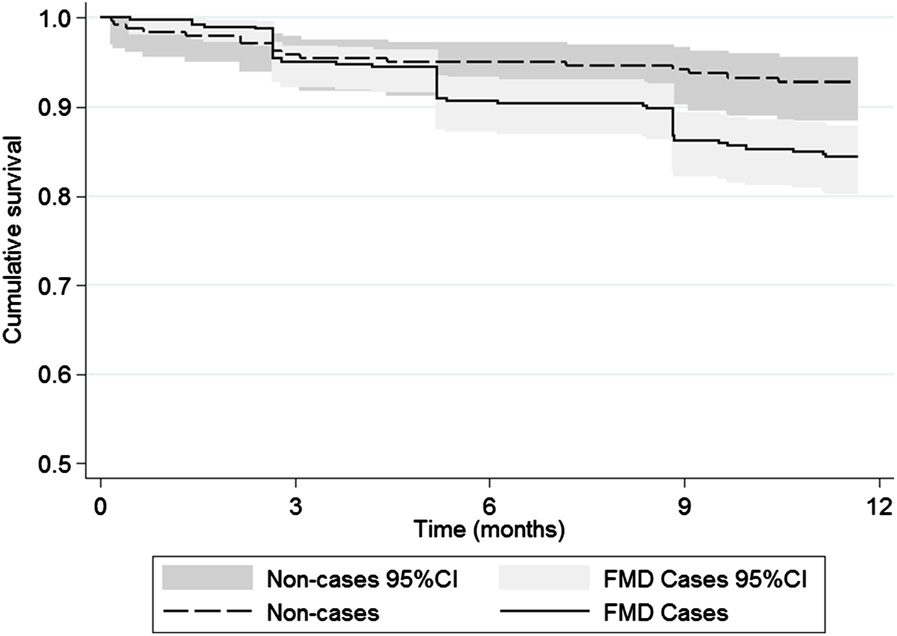
**Unadjusted Kaplan-Meier survival curves for FMD cases and non-cases related to culling.** Animals were included in the analysis if present on the farm during the outbreak period (31^st^ August-28^th^ September 2012) and were followed for 12 months after the commencement of the outbreak. Culling was defined as exiting the herd due to any disease, death or low production. Log-rank test for equality of survivor function, *P* = 0.0036. The Y-axis represents the cumulative probability of not being culled.

Examination of Kaplan-Meier plots showed differences between FMD cases and non-cases for both outcomes with strong statistical evidence for a difference provided by the log rank tests for culling alone (Figure 1, Figure 2). For clinical mastitis, it can be seen among FMD cases that there is a large increase in hazard in the 1–2 months after the outbreak. Thereafter the hazard ratio appears equivalent to that in non-cases and then becomes higher among non-cases seven months after the outbreak started although the confidence intervals overlap (Figure 1). For culling, the hazards grow progressively apart with animals appearing to exit in groups at 3, 5 and 9 months after the beginning of the follow-up period (Figure 2). After nine months the confidence intervals do not overlap.

As part of the assessment into whether age, parity, breed, lactation stage and previous disease events confounded the association between the primary risk factor and outcomes in this study, univariable associations between these factors were analysed. Previous analysis of the entire study population (*n* = 644) indicated that older animals and those with a more exotic breed pedigree were at increased risk of reported clinical FMD [25]. Further analysis in this study indicated an association between cattle experiencing eye disease, lameness, diarrhoea or tick-borne disease in the 12 months preceding the outbreak and the risk of clinical FMD (Additional file 1). Older cows in later stages of lactation had an increased rate of culling (Table 2). This outcome was also associated with dystocia, clinical mastitis, tick-borne disease and having any disease event in the previous 12 months (Additional file 1). Univariable analysis of the subpopulation considered at risk of clinical mastitis revealed older cows in later stages of lactation had an increased rate of this outcome, which was also the case for cows with increasing indigenous breed pedigrees (Table 3). Previous lameness was also associated with increased mastitis incidence rate (Additional file 2). In the final multivariable models that were adjusted for age, the only disease event that was included was having tick-borne disease as this confounded the association between FMD and culling.

**Table 2:** **Culling - characteristics of the study population (n = 644) and univariable analysis**

**Variable**	**Total population**		**Culling**		
	**N**	**(col %)**	**Rate per 1000 cattle-months (95% CI)**	**HR (95% CI)**	***P*** **-value**
**FMD**					
Yes	400	62.1	14.4 (11.2, 18.6)	2.2 (1.3, 3.7)	0.005
No	244	37.9	6.6 (4.1, 10.6)		
**Age (quintiles)** ^**a**^					
−28- <227d	128	19.9	5.0 (2.4, 10.5)	1.6^b^ (1.3, 1.9)	<0.0001
227- < 577d	129	20.0	2.0 (0.66, 6.3)		
577- < 974d	129	20.0	12.6 (7.8, 20.3)		
974- < 1363d	129	20.0	17.1 (11.2, 25.9)		
1364-3543d	129	20.0	23.2 (15.9, 33.8)		
**Parity**					
0	381	59.2	4.8 (3.1, 7.4)	1.5^b^ (1.3, 1.8)	<0.0001
1	138	21.4	27.1 (19.5, 37.8)		
2	77	12.0	11.6 (6.1, 22.4)		
3	29	4.5	36.8 (19.2, 70.7)		
≥4	19	3.0	18.2 (5.9, 56.6)		
**Days in milk**					
Pre-lactating	373	57.9	4.9 (3.1, 7.6)	1.7^b^ (1.5, 1.9)	<0.0001
Early-lactation (<0-100d)	107	16.6	15.2 (9.3, 24.9)		
Mid-lactation (101-250d)	85	13.2	18.8 (11.3, 31.2)		
Late-lactation (>250d)	44	6.8	25.4 (13.7, 47.2)		
Dry	35	5.4	46.0 (27.7, 76.3)		
**Breed**					
100% Exotic breed	551	85.6	11.5 (9.0, 14.6)	0.7^b^ (0.6, 1.3)	0.52
<25% Indigenous	53	8.2	14.6 (7.3, 29.2)		
25% Indigenous	28	4.4	3.7 (0.5, 26.0)		
50% indigenous	12	1.9	8.8 (1.2, 62.7)		

^a^Negative values reflect animals born during the outbreak period ^b^Included as linear variables based on likelihood ratio tests.

Univariable analysis examines putative associations with the primary outcome (culling) for cattle present during an outbreak of foot-and-mouth disease (FMD) on a dairy farm in Nakuru County, Kenya. Culling is defined as exiting the herd due to any disease or death. HR = Hazard ratio.

**Table 3:** **Clinical mastitis - characteristics of the study population (** ***n =*** **409) and univariable analyses**

**Variable**			**FMD**		**Clinical mastitis**		
	**N**	**(col %)**	**N (row %)**	***P*** **-value**	**Rate per 1000 cattle-months (95% CI)**	**HR (95% CI)**	***P*** **-value**
**FMD**							
Yes	323	79.0	-	-	18.0 (13.7, 23.7)	1.3 (0.68, 2.5)	0.43
No	86	21.0	-		13.8 (7.6, 24.9)		
**Age (quintiles)**							
1.5- < 2.0y	81	19.8	54 (66.7)	0.0073^a^	3.4 (1.1, 10.6)	1.8^b^ (1.5, 2.2)	<0.0001
2.0- < 2.8y	82	20.1	61 (74.4)		7.5 (3.3, 16.6)		
2.8- < 3.5y	82	20.1	71 (86.6)		17.8 (10.3, 30.7)		
3.5- < 4.3y	83	20.3	73 (88.0)		18.1 (10.5, 31.2)		
4.3-9.7y	81	19.8	64 (79.0)		50.8 (35.1, 73.6)		
**Parity**							
0	146	35.7	107 (73.3)	0.042^a^	1.9 (0.6, 6.0)	1.7 ^b^ (1.4, 2.1)	<0.0001
1	138	33.7	110 (79.7)		22.4 (15.3, 32.9)		
2	77	18.8	67 (87.0)		33.2 (21.6, 50.9)		
3	29	7.1	22 (75.9)		29.1 (13.1, 64.9)		
≥4	19	4.7	17 (89.5)		55.6 (26.5, 126.0)		
**Days in milk** ^**c**^							
Pre-lactating	138	33.7	102 (73.9)	0.027	1.4 (0.34, 5.4)	1.7 ^b^ (1.4, 2.0)	<0.0001
Early-lactation (<0-100d)	107	26.2	88 (82.2)		18.5 (11.5, 29.7)		
Mid-lactation (101-250d)	85	20.8	75 (88.2)		27.7 (17.7, 43.5)		
Late-lactation (>250d)	44	10.8	35 (79.6)		45.2 (26.8, 76.3)		
Dry	35	8.6	23 (65.7)		37.8 (20.9, 68.2)		
**Breed**							
100% Exotic breed	351	85.8	285 (81.2)	0.0046^a^	15.7 (11.9, 20.7)	1.3 ^b^ (1.0, 1.8)	0.053
<25% Indigenous	27	6.6	20 (74.1)		23.5 (10.6, 52.3)		
25% Indigenous	19	4.7	10 (52.6)		12.8 (3.2, 51.1)		
50% indigenous	12	2.9	8 (66.7)		54.8 (22.8, 131.6)		

^a^Chi-square test for trend.

^b^Included as linear variables based on likelihood ratio tests ^c^Defined at the beginning of the outbreak period. Univariable analyses examine the associations with the primary risk factor (being a case of clinical FMD) and primary outcome (clinical mastitis) for cattle present during an outbreak of foot-and-mouth disease (FMD) on a dairy farm in Nakuru County, Kenya. Cattle are included in these analyses if over the age of 18 months at the start of outbreak, considered as the age when clinical mastitis becomes a possibility. HR = Hazard ratio.

Global Schoenfeld residual tests performed prior to backward model fitting revealed strong evidence of a departure from the proportional hazards assumption in both mastitis and culling models (Additional file 3). In the mastitis model, tests for individual variables and an examination of the scaled Schoenfeld residual plot showed the FMD variable to be responsible for most of this deviation particularly in the two month period after the onset of the outbreak. This lack of proportionality is consistent with the Kaplan-Meier (Figure 1) and Nelson-Aalen plots. AIC tests indicated a logarithmic multiplier function to provide the best model fit when the FMD variable was included as a time-varying effect which led to a more stable scaled Schoenfeld residual plot and decreased the global test statistic. In the culling model, there was strong evidence that the lactation stage showed significant departure from the proportional hazards assumption which was similarly rectified by the inclusion of a logarithmic multiplier function with time based on AIC tests (Additional file 3).

**Figure 3:**
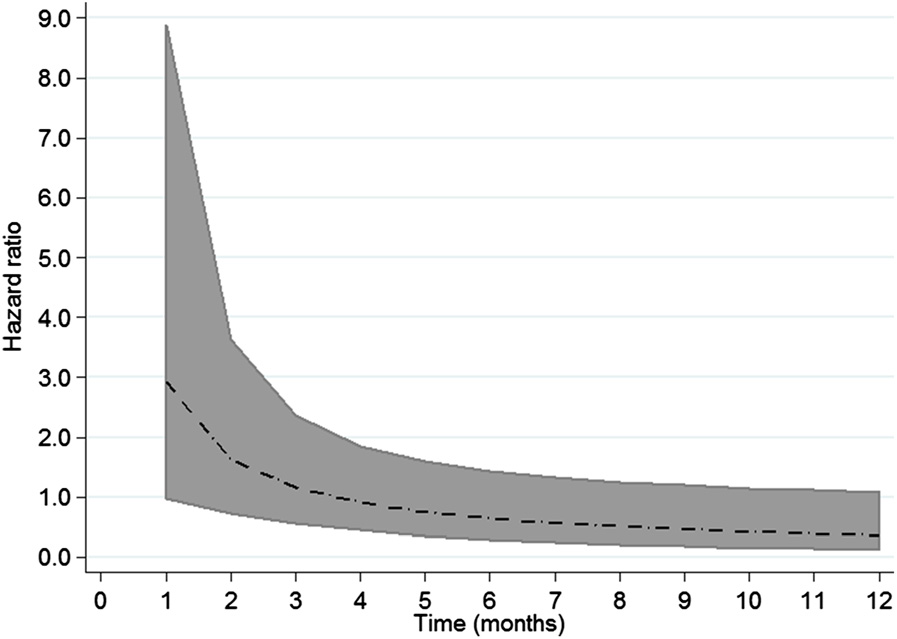
**Variation in hazard ratio over time for cases of FMD developing clinical mastitis.** Animals were included in the analysis if present on the farm during the outbreak period (31^st^ August-28^th^ September 2012) and were followed for 12 months after the commencement of the outbreak. Cattle were included if over the age of 18 months at the start of outbreak, considered as the age when clinical mastitis becomes a possibility.

The final multivariable model for mastitis incorporating the time-varying effect of FMD was also adjusted for age, lactation stage, and breed (Table 4). There was evidence that the hazard ratio was significantly greater than 1.0, one month after the beginning of the outbreak with the effect disappearing in the subsequent follow up period as confidence intervals continually overlap with 1.0 (Figure 3). For culling, the estimate was adjusted for age, parity, and the presence of tick-borne disease in the previous 12 months with lactation stage as the time varying effect (Table 4). Although there was a trend for cattle affected by FMD to be culled sooner during the 12 month follow-up period, the statistical evidence was weak (HR = 1.7, 95% CI 0.9-3.1, *P* = 0.12).

**Table 4:** **Final multivariable Cox-regression model examining the association of FMD with clinical mastitis and culling**

**Variable**	**Clinical mastitis**			**Culling**		
		**HR (95%CI)**	***P*** **-value**		**HR (95% CI)**	***P*** **-value**
**FMD**						
Case		2.9 (0.97, 8.9)	0.057		1.7 (0.88, 3.1)	0.12
Non-case		Baseline	-		Reference	-
**Age** ^**a**^Age category Age category						
	1.5- < 2.0 y	Baseline	-	−28- <227d	Baseline	-
	2.0- < 2.8 y	0.61 (0.12, 3.0)	0.54	227- < 577d	0.29 (0.07, 1.2)	0.090
	2.8- < 3.5 y	0.77 (0.16, 3.7)	0.75	577- < 974d	0.93 (0.32, 2.7)	0.89
	3.5- < 4.3 y	0.64 (0.12, 3.3)	0.60	974- < 1363d	0.76 (0.21, 2.7)	0.76
	4.3-9.7 y	2.6 (0.45, 15.1)	0.29	1364-3543d	1.5 (0.34, 6.3)	0.60
**Lactation stage**					0.45 (0.24, 0.83)	0.010
Non-lactating		-	-			
Early lactation (<0-100d)		-	-			
Mid lactation (101-250d)		-	-			
Late lactation (>250d)		-	-			
Dry		-	-			
**Breed** ^b^		1.4	0.031		-	-
**Tick borne disease (last 12 months)**						
Yes		-	-		3.2 (1.8, 5.9)	<0.0001
No		-	-		Baseline	
**Parity**						
0		Baseline	-		Baseline	
1		11.3 (2.5, 51.2)	0.002		2.8 (0.91, 8.4)	0.074
2		9.8 (1.7, 55.9)	0.010		0.66 (0.16, 2.6)	0.55
3		4.6 (0.65, 32.6)	0.13		2.4(0.60, 9.8)	0.21
≥4		8.1 (1.2, 55.8)	0.033		0.88 (0.16, 5.0)	0.89
***Time varying interactions***						
**FMD**		0.43	0.016		-	-
**Lactation stage**		-	-		2.0 (1.4, 2.6)	<0.0001

^a^Age categories based on quintiles.

^b^Included as a linear effect. Categories are 100% exotic breed, <25% indigenous, 25% indigenous, 50% indigenous. Animals were included in the analysis if present on the farm during the outbreak period (31^st^ August-28^th^ September 2012) and were followed for 12 months after the commencement of the outbreak. For the analysis of clinical mastitis, cattle were included if over the age of 18 months at the start of outbreak, considered as the age when clinical mastitis becomes a possibility. Culling was defined as exiting the herd due to any disease, death of low production. Hazard ratios (HR) incorporate time varying effects with logarithmic multiplier functions to account for non-proportional hazards. Final model for mastitis included age, breed and parity with FMD incorporated as a time varying effect. For culling the final model included age, lactation stage, tick-borne disease in the 12 months preceding the outbreak and parity with lactation stage as a time varying effect.

## Discussion

During this outbreak on a large-scale dairy farm in Kenya, 400/644 (62.1%) of cattle were reported to be affected with FMD due to serotype SAT2. The outbreak lasted for 29 days with the index case identified on 31^st^ August 2012 and the last case on the 28^th^ September 2012. In the 12-month follow up period commencing on the day the index case was identified, 76 were culled or died. For cattle aged 18 months or greater at the start of the outbreak, 63 developed clinical mastitis. Although in the univariable analysis FMD cases tended to be culled sooner than non-cases after the outbreak onset, after adjustment for possible confounders there was weak evidence to support this observation. In contrast, for clinical mastitis, univariable analysis showed no effect of FMD on rate of mastitis but after adjusting for the time varying effect of being a case there was good evidence of an increased rate in the first month after the onset of the outbreak.

For correct interpretation of the Kaplan-Meier curves for clinical mastitis and culling, one must consider the expected timing of disease impact and farm management. The association between FMD and clinical mastitis is related mainly to the FMD lesions that develop on the teats which is likely to have increased the susceptibility to secondary bacterial infection [26]. As the bacteria are likely to be mainly environmental in origin, milk culture results would support this hypothesis but were not performed in this outbreak. Since infection is likely to occur soon after the appearance of lesions, this explains the increased hazard among FMD cases in the early stages of the follow-up period. The reason behind what appears to be an increased mastitis rate in non-FMD cases that was seen several months after the outbreak is unknown but this difference was not statistically significant (Figure 1). In contrast, the rate of cows exiting the herd due to culling appears to increase throughout the follow-up period (Figure 2). This is because FMD typically has a low mortality rate and farm exit will generally occur once a cow has ceased producing milk and after an appropriate period of time to fatten. Following this logic, the overall effect of any non-fatal disease on culling rate will only become apparent around a year later when cows which were in early lactation at the outset of the outbreak reach the end of their lactation (presuming they continue to lactate after recovering from the disease). Lactation stage has been previously shown to be associated with culling in a survival analysis study of Holstein-Friesian cattle in Kenya, with cows in later lactation more likely to be culled than those in early lactation [27]. As a consequence, lactation stage at outbreak onset was included as a time-varying effect in the multivariable model. The decreases seen in the culling survival curve at around 3, 5 and 9 months are probably due to management issues as cattle are removed from the farm in groups once a decision has been made to cull.

Despite the Kaplan-Meier curve and log-rank test revealing a trend indicating increased culling with clinical cases of FMD (Figure 2), in the multivariable model there was only very weak statistical evidence of an association. In this outbreak, older cows appeared at greater risk of FMD and due to their advanced age, were at increased risk of culling. Therefore age was a strong confounder of the association between being a case of FMD and being culled. In contrast, the effect of FMD on clinical mastitis was strong enough that even after adjustment for age there was still a pronounced effect in the early follow-up period.

Animals that had suffered from a tick-borne disease (TBD) prior to the FMD outbreak were more likely to develop FMD and also to be culled compared to those that had no TBD (Additional file 1). This confounder was present after adjusting for age which has been shown to be associated with the incidence of TBD [17]. Tick-borne diseases (theileriosis (East Coast Fever, ECF), babesiosis and anaplasmosis) have been identified as a major cause of cow death and culling among smallholder dairy farms in Kenya and neighbouring Tanzania [28,29]. The farm does not consistently record which disease was encountered hence they were all included as one disease condition. Although no definitive diagnosis of the condition was made, the clinical signs associated with these conditions are easily observed and commonly encountered. In Kenya, ECF has been identified as the disease with the highest impact on livelihoods among pastoralists, marginally ahead of FMD [30]. The impact of ECF on culling has been less well characterised on large-scale dairy farms although an outbreak on a large-scale farm in Tanzania due to a breakdown in dipping regime led to severe economic losses [31]. Acaricide dips are in wide use among large-scale farms in Kenya. It has been anecdotally suggested that previous exposure to other infectious diseases may increase the susceptibility to FMD although this is the first study to the authors’ knowledge that provides evidence for such an association.

This study has measured “real-life” impact of a FMD outbreak under field conditions. The majority of cattle present on the farm were affected with FMD despite vaccine being given to all cattle approximately every 4 months. Although previous analysis found no evidence of vaccine effectiveness in preventing clinical disease [25], it cannot be ruled out that the vaccine provided some protection leading to an underestimate of the impact if the outbreak had occurred in an immunologically naive herd. Vaccination for FMD is common among large-scale herds in Kenya, making these results relevant to this population and useful when considering the impact of a vaccination programme. However, due to variable degrees of protection that the vaccine may afford, it is likely that FMD effects will vary considerably among large-scale herds. Similar estimates from outbreaks on other dairy farms with other serotypes would therefore be useful to demonstrate the range of impacts in the field.

The study was on a large-scale dairy farm. As such farms are in the minority among dairy farms in Kenya, care must be taken when generalising these results to the broader dairy cow population. There are, however, similarities to the local smallholder cattle population that need emphasising. The breeds of cattle on the study farm are predominantly exotic (mainly Holstein-Friesian) with some indigenous cross-breeding. Among smallholder dairy farms in Kenya, Holstein-Friesian is also the most common breed due to the higher milk yields [32]. Indeed the study farm does sell cattle to local smallholder farmers. Additionally, the nutritional management on the study farm is similar to that employed in organised smallholder dairy regions where farmers have access to concentrate feeds, as is the case in the study area.

Data from surrounding affected and non-affected smallholder farms would add weight to these findings. The routine recording of data on production parameters meant the data were easily extracted and analysed in this study. Such routine recording of data is rarely performed on smallholder farms making similar studies more difficult. Experience from the field reveals that smallholder farmers tend to use less FMD vaccine than the larger farms, so the impact on mastitis and culling may be higher. Overall, it would not be surprising to see a similar impact among smallholder dairy cattle although the overall socio-economic impact is likely to be different in these production systems. The results of this study may be used in relevant economic models for FMD, although more research is needed to estimate the cost of mastitis in Kenyan settings so that cost-effectiveness of control programmes may be described.

The current study has raised several methodological issues. The statistical modelling for culling suggested that including age and parity improved model fit. There is strong collinearity between these two variables. Since the objective of the study is to look only at the impact of FMD on the primary outcomes, it was decided to include both of these covariates although this restricts the interpretation of their associated effect estimates in the final multivariable regression model.

Survival analysis utilising a Cox proportional hazards regression model relies upon the fundamental assumption that the hazards for comparison groups are proportional over the follow-up period [33]. If a model covariate has different effects at different time periods, this can violate this assumption and lead to invalid statistical associations. Despite the importance of this assumption many published studies do not provide evidence of the assessment. In one review of clinical trials for human cancer, only 5/64 studies included any form of test for proportional hazards [34]. The importance of ensuring the validity of the assumption was particularly clear in this study as adjusting for a time varying effect led to a large difference in the effect of FMD on the two primary outcomes.

In conclusion, this study is the first to utilise survival analysis methods to estimate the effect of FMD on subsequent clinical mastitis and culling. These results offer a detailed assessment of disease impact that can inform future cost analyses that are currently over-reliant on expert opinion, assumptions and limited use of field data. It is only through performing such studies in different settings that the real impact of FMD can be estimated in endemic countries and inform the cost-effectiveness of national and international disease control programmes.

## Additional files

Additional file 1:
**Culling – univariable associations with other diseases.** Previous disease experienced in the 12 months prior to the commencement of the outbreak and the association with being a case of FMD and culling rate. For dystocia and retained foetal membranes, the population at risk was all animals that had given birth in the 12 months prior to the outbreak. For abortions, animals were considered a risk at the commencement of normal age at first service (16 months). For clinical mastitis, the population at risk was animals over the age of 18 months. Hazard ratios are calculated using Cox regression. Additional file 2:
**Clinical mastitis - univariable associations with other diseases.** Previous disease experienced in the 12 months prior to the commencement of the outbreak and the association with being a case of FMD and mastitis rate. For dystocia and retained foetal membranes, the population at risk was all animals that had given birth in the 12 months prior to the outbreak. For abortions, animals were considered a risk at the commencement of normal age at first service (16 months). Hazard ratios are calculated using Cox regression. Additional file 3:
**Tests for non-proportionality.** Schoenfeld residual tests for non-proportionality of each indicator variable prior to model backward fitting. For mastitis, the initial model included FMD, age, parity, lactation stage and breed. For culling, the initial model included FMD, age, parity, lactation stage, tick-borne disease in the previous 12 months, clinical mastitis in the previous 12 months, and having any reported disease in the previous 12 months.
