# Supplementary material for: Impact of foot-and-mouth disease on mastitis and culling on a large-scale dairy farm in Kenya
Source: Vet Res. 2015 Apr 16;46(1):41. doi: 10.1186/s13567-015-0173-4 (PMC4397692; doi:10.1186/s13567-015-0173-4)
Supplement: Additional file 1: — Culling – univariable associations with other diseases. Previous disease experienced in the 12 months prior to the commencement of the outbreak and the association with being a case of FMD and culling rate. For dystocia and retained foetal membranes, the population at risk was all animals that had given birth in the 12 months prior to the outbreak. For abortions, animals were considered a risk at the commencement of normal age at first service (16 months). For clinical mastitis, the population at risk was animals over the age of 18 months. Hazard ratios are calculated using Cox regression. [file 13567_2015_173_MOESM1_ESM.docx]

| **Disease event** | **N** | **Col %** | **FMD** |  | **Culling** |  |  |
| --- | --- | --- | --- | --- | --- | --- | --- |
|  |  |  | N (row %) | *P*-value | Rate per 1000 cattle-months (95%CI) | HR (95%CI) | *P*-value ^b^ |
| **Abortion** |  |  |  |  |  |  |  |
| Yes | 16 | 3.9 | 14 (87.5) | 0.54^a^ | 25.0 (9.4, 66.5) | 1.5 (0.56, 4.2) | 0.41 |
| No | 393 | 96.1 | 309 (78.6) |  | 16.2 (12.6, 20.7) |  |  |
|  |  |  |  |  |  |  |  |
| **Abscess** |  |  |  |  |  |  |  |
| Yes | 6 | 0.9 | 3 (50.0) | 0.54 | 0 (-) | - | - |
| No | 638 | 99.1 | 397 (62.2) |  | 11.5 (9.2, 14.4) |  |  |
|  |  |  |  |  |  |  |  |
| **Broken leg** |  |  |  |  |  |  |  |
| Yes | 1 | 0.2 | 1 (100.0) | 0.99 ^a^ | 0(-) | - | - |
| No | 643 | 99.8 | 399 (62.1) |  | 11.4 (9.1, 14.3) |  |  |
|  |  |  |  |  |  |  |  |
| **Dystocia** |  |  |  |  |  |  |  |
| Yes | 4 | 2.1 | 2 (50.0) | 0.11^a^ | 84.0 (21.0, 336.0) | 4.8 (1.2, 20.3) | 0.031 |
| No | 184 | 97.9 | 157 (85.3) |  | 17.2 (12.0, 24.5) |  |  |
|  |  |  |  |  |  |  |  |
| **Eye disease** |  |  |  |  |  |  |  |
| Yes | 114 | 17.7 | 84 (73.7) | 0.005 | 11.0 (9.0, 14.7) | 1.0 (0.53, 1.7) | 0.90 |
| No | 530 | 82.3 | 316 (59.6) |  | 11.5 (9.0, 14.7) |  |  |
|  |  |  |  |  |  |  |  |
| **Lameness** |  |  |  |  |  |  |  |
| Yes | 40 | 6.2 | 32 (80.0) | 0.016 | 9.8 (3.7, 26.1) | 0.9 (0.3, 2.3) | 0.75 |
| No | 604 | 93.8 | 368 (60.9) |  | 11.5 (9.1, 14.5) |  |  |
|  |  |  |  |  |  |  |  |
| **Dislocated hip** |  |  |  |  |  |  |  |
| Yes | 1 | 0.2 | 1 (100.0) | 0.99 ^a^ | 0(-) | - | - |
| No | 643 | 99.8 | 399 (62.1) |  | 11.4 (9.1, 14.3) |  |  |
|  |  |  |  |  |  |  |  |
| **Clinical mastitis** |  |  |  |  |  |  |  |
| Yes | 35 | 8.6 | 29 (82.9) | 0.56 | 32.9 (17.7, 61.2) | 2.2 (1.1, 4.2) | 0.025 |
| No | 609 | 91.4 | 294 (78.6) |  | 15.2 (11.7, 19.7) |  |  |
|  |  |  |  |  |  |  |  |
| **Pneumonia** |  |  |  |  |  |  |  |
| Yes | 22 | 3.4 | 13 (59.1) | 0.77 | 12.3 (4.0, 38.2) | 1.1 (0.34, 3.4) | 0.89 |
| No | 622 | 96.6 | 387 (62.2) |  | 11.4 (9.0, 14.3) |  |  |
|  |  |  |  |  |  |  |  |
| **Retained foetal membranes** |  |  |  |  |  |  |  |
| Yes | 4 | 2.1 | 4 (100.0) | 0.99^a^ | 28.1 (4.0, 199.2) | 1.5 (0.21, 11.1) | 0.68 |
| No | 184 | 97.5 | 155 (84.2) |  | 17.8 (12.6, 25.4), |  |  |
|  |  |  |  |  |  |  |  |
| **Diarrhoea** |  |  |  |  |  |  |  |
| Yes | 49 | 7.6 | 17 (34.7) | <0.0001 | 5.5 (1.8, 17.1) | 0.46 (0.15, 1.5) | 0.18 |
| No | 595 | 92.4 | 383 (64.4) |  | 11.9 (9.5, 15.0) |  |  |
|  |  |  |  |  |  |  |  |
| **Snake bite** |  |  |  |  |  |  |  |
| Yes | 1 | 0.2 | 0 (0) | 0.38 ^a^ | 0 (-) | - | - |
| No | 643 | 99.8 | 400 (62.2) |  | 11.4 (9.1, 14.3) |  |  |
|  |  |  |  |  |  |  |  |
| **Three-day sickness** |  |  |  |  |  |  |  |
| Yes | 3 | 0.5 | 63 (100.0) | 0.18 | 0 (-) | - | - |
| No | 641 | 99.5 | 397 (61.9) |  | 11.4 (9.1, 14.3) |  |  |
|  |  |  |  |  |  |  |  |
| **Tick-borne disease** |  |  |  |  |  |  |  |
| Yes | 46 | 7.1 | 36 (78.3) | 0.019 | 38.0 (22.9, 63.0) | 3.9 (2.2, 6.9) | <0.0001 |
| No | 596 | 92.9 | 364 (60.9) |  | 9.7 (7.6, 12.5) |  |  |
|  |  |  |  |  |  |  |  |
| **Vulval discharge** |  |  |  |  |  |  |  |
| Yes | 8 | 1.2 | 7 (87.5) | 0.14 | 26.5 (6.6, 106.1) | 2.4 (0.59, 9.6) | 0.22 |
| No | 636 | 98.8 | 393 (61.8) |  | 11.2 (8.9, 14.1) |  |  |
|  |  |  |  |  |  |  |  |
| **Wound** |  |  |  |  |  |  |  |
| Yes | 17 | 2.6 | 7 (41.2) | 0.071 | 0 (-) | 0.5 (0.06, 3.3) | 0.42 |
| No | 627 | 97.4 | 393 (62.7) |  | 11.6 (9.2, 14.5) |  |  |
|  |  |  |  |  |  |  |  |
| **Any disease** |  |  |  |  |  |  |  |
| Yes | 269 | 41.8 | 185 (68.8) | 0.003 | 15.5 (11.4, 21.0) | 1.8 (1.1, 2.8) | 0.0095 |
| No | 375 | 58.2 | 215 (57.3) |  | 8.6 (6.1, 12.0) |  |  |

^a^ Fisher’s exact test ^b^ Likelihood ratio test
