# Supplementary material for: Impact of foot-and-mouth disease on mastitis and culling on a large-scale dairy farm in Kenya
Source: Vet Res. 2015 Apr 16;46(1):41. doi: 10.1186/s13567-015-0173-4 (PMC4397692; doi:10.1186/s13567-015-0173-4)
Supplement: Additional file 2: — Clinical mastitis - univariable associations with other diseases. Previous disease experienced in the 12 months prior to the commencement of the outbreak and the association with being a case of FMD and mastitis rate. For dystocia and retained foetal membranes, the population at risk was all animals that had given birth in the 12 months prior to the outbreak. For abortions, animals were considered a risk at the commencement of normal age at first service (16 months). Hazard ratios are calculated using Cox regression. [file 13567_2015_173_MOESM2_ESM.docx]

| **Disease event** | **N** | **Col %** | **FMD** |  | **Clinical mastitis** |  |  |
| --- | --- | --- | --- | --- | --- | --- | --- |
|  |  |  | N (row %) | *P*-value | Rate per 1000 cattle-months (95%CI) | HR (95%CI) | *P*-value^b^ |
| **Abortion** |  |  |  |  |  |  |  |
| Yes | 16 | 3.9 | 14 (87.5) | 0.39 | 6.3 (0.9, 44.7) | 0.37 (0.05, 2.7) | 0.24 |
| No | 393 | 96.1 | 309 (78.6) |  | 17.6 (13.7, 22.6) |  |  |
|  |  |  |  |  |  |  |  |
| **Abscess** |  |  |  |  |  |  |  |
| Yes | 5 | 1.2 | 2 (40.0) | 0.031 | 0 (-) | - | - |
| No | 404 | 98.8 | 321 (79.5) |  | 17.4 (13.6, 22.2) |  |  |
|  |  |  |  |  |  |  |  |
| **Broken leg** |  |  |  |  |  |  |  |
| Yes | 0 | 0.0 | - | - | 0 (-) | - | - |
| No | 409 | 100.0 | 323 (79.0) |  | 17.1 (13.4, 21.9) |  |  |
|  |  |  |  |  |  |  |  |
| **Dystocia** |  |  |  |  |  |  |  |
| Yes | 4 | 2.1 | 2 (50.0) | 0.11^a^ | 44.3 (6.2, 314.7) | 1.2 (0.17, 8.9) | 0.85 |
| No | 184 | 97.9 | 157 (85.3) |  | 28.7 (21.2, 38.8) |  |  |
|  |  |  |  |  |  |  |  |
| **Eye disease** |  |  |  |  |  |  |  |
| Yes | 80 | 19.6 | 59 (73.8) | 0.20 | 22.8 (14.0, 37.2) | 1.4 (0.82, 2.6) | 0.22 |
| No | 329 | 80.4 | 264 (80.2) |  | 15.8 (11.8, 21.0) |  |  |
|  |  |  |  |  |  |  |  |
| **Lameness** |  |  |  |  |  |  |  |
| Yes | 31 | 7.6 | 26 (83.9) | 0.49 | 37.6 (19.6, 72.2) | 2.3 (1.1, 4.6) | 0.024 |
| No | 378 | 92.4 | 297 (78.6) |  | 15.7 (12.0, 20.5) |  |  |
|  |  |  |  |  |  |  |  |
| **Dislocated hip** |  |  |  |  |  |  |  |
| Yes | 1 | 0.2 | 1 (100.0) | 0.61 | 0 (-) | - | - |
| No | 408 | 99.8 | 322 (78.9) |  | 17.2 (13.4, 22.0) |  |  |
|  |  |  |  |  |  |  |  |
| **Clinical mastitis** |  |  |  |  |  |  |  |
| Yes | 35 | 8.6 | 29 (82.9) | 0.56 | 22.1 (9.9, 49.1) | 1.2 (0.53, 2.9) | 0.64 |
| No | 374 | 91.4 | 294 (78.6) |  | 16.7 (12.9, 21.7) |  |  |
|  |  |  |  |  |  |  |  |
| **Pneumonia** |  |  |  |  |  |  |  |
| Yes | 13 | 3.2 | 10 (76.9) | 0.85 | 22.7 (7.3, 70.5) | 1.4 (0.44, 4.4) | 0.59 |
| No | 396 | 96.8 | 313 (79.0) |  | 16.9 (13.1, 21.8) |  |  |
|  |  |  |  |  |  |  |  |
| **Retained foetal membranes** |  |  |  |  |  |  |  |
| Yes | 4 | 2.1 | 4 (100.0) | 0.99^a^ | 68.9 (17.2, 275.6) | 2.5 (0.60, 10.3) | 0.27 |
| No | 184 | 97.9 | 155 (84.2) |  | 28.1 (20.7, 38.2) |  |  |
|  |  |  |  |  |  |  |  |
| **Diarrhoea** |  |  |  |  |  |  |  |
| Yes | 3 | 0.7 | 2 (66.7) | 0.60 | 0 (-) | - | - |
| No | 406 | 99.3 | 321 (79.1) |  | 17.3 (13.5, 22.1) |  |  |
|  |  |  |  |  |  |  |  |
| **Snake bite** |  |  |  |  |  |  |  |
| Yes | 1 | 0.2 | 0 (0.0) | 0.052 | 0 (-) | - | - |
| No | 409 | 99.8 | 323 (79.2) |  | 17.2 (13.4, 22.0) |  |  |
|  |  |  |  |  |  |  |  |
| **Three-day sickness** |  |  |  |  |  |  |  |
| Yes | 3 | 0.7 | 3 (100.0) | 0.37 | 0 (-) | - | - |
| No | 406 | 99.3 | 320 (78.8) |  | 17.2 (13.5, 22.1) |  |  |
|  |  |  |  |  |  |  |  |
| **Tick-borne disease** |  |  |  |  |  |  |  |
| Yes | 42 | 10.3 | 35 (83.3) | 0.46 | 29.7 (15.4, 57.0) | 1.7 (0.83, 3.4) | 0.18 |
| No | 367 | 89.7 | 288 (78.5) |  | 16.0 (12.2, 20.9) |  |  |
|  |  |  |  |  |  |  |  |
| **Vulval discharge** |  |  |  |  |  |  |  |
| Yes | 8 | 2.0 | 7 (87.5) | 0.55 | 31.1 (7.8, 124.3) | 1.7 (0.42, 7.0) | 0.49 |
| No | 401 | 98.0 | 316 (78.8) |  | 16.9 (13.1, 21.7) |  |  |
|  |  |  |  |  |  |  |  |
| **Wound** |  |  |  |  |  |  |  |
| Yes | 8 | 2.0 | 5 (62.5) | 0.25 | 11.9 (1.7, 84.6) | 0.74 (0.10, 5.3) | 0.75 |
| No | 401 | 98.0 | 318 (79.3) |  | 17.2 (13.4, 22.1) |  |  |
|  |  |  |  |  |  |  |  |
| **Any disease** |  |  |  |  |  |  |  |
| Yes | 185 | 45.2 | 145 (78.4) | 0.79 | 19.4 (13.7, 27.6) | 1.2 (0.75, 2.0) | 0.41 |
| No | 224 | 54.8 | 178 (79.5) |  | 15.3 (10.8, 21.7) |  |  |

^a^ Fisher’s exact test ^b^ Likelihood ratio test
