# Supplementary material for: Impact of foot-and-mouth disease on mastitis and culling on a large-scale dairy farm in Kenya
Source: Vet Res. 2015 Apr 16;46(1):41. doi: 10.1186/s13567-015-0173-4 (PMC4397692; doi:10.1186/s13567-015-0173-4)
Supplement: Additional file 3: — Tests for non-proportionality. Schoenfeld residual tests for non-proportionality of each indicator variable prior to model backward fitting. For mastitis, the initial model included FMD, age, parity, lactation stage and breed. For culling, the initial model included FMD, age, parity, lactation stage, tick-borne disease in the previous 12 months, clinical mastitis in the previous 12 months, and having any reported disease in the previous 12 months. [file 13567_2015_173_MOESM3_ESM.docx]

| **Variable** | **Clinical mastitis** | | **Culling** | |
| --- | --- | --- | --- | --- |
|  | χ^2^ statistic | *P*-value | χ^2^ statistic | *P*-value |
| FMD | 2.0 | 0.16 | 1.0 | 0.31 |
| Age (quintiles) |  |  |  |  |
| Quintile 1 | - | - | - | - |
| Quintile 2 | 0.87 | 0.35 | 0.5 | 0.50 |
| Quintile 3 | 3.1 | 0.078 | 2.5 | 0.11 |
| Quintile 4 | 3.7 | 0.054 | 0.00 | 0.98 |
| Quintile 5 | 3.0 | 0.089 | 0.00 | 0.98 |
| Parity |  |  |  |  |
| 0 | - | - | - | - |
| 1 | 0.37 | 0.54 | 0.08 | 0.78 |
| 2 | 0.25 | 0.62 | 0.03 | 0.86 |
| 3 | 0.47 | 0.50 | 0.42 | 0.52 |
| 4+ | 0.35 | 0.55 | 0.13 | 0.72 |
| Lactation stage ^a^ |  |  | 11.4 | 0.0008 |
| Non-lactating | - | - |  |  |
| Early lactation (<0-100d) | 0.53 | 0.46 |  |  |
| Mid lactation (101-250d) | 0.03 | 0.86 |  |  |
| Late lactation (>250d) | 0.07 | 0.79 |  |  |
| Dry | 0.11 | 0.74 |  |  |
| Tick bone disease ^b^ | - | - | 2.1 | 0.18 |
| Clinical mastitis ^b^ | - | - | 3.2 | 0.072 |
| Breed | 1.5 | 0.23 |  |  |
| Any disease ^b^ |  |  | 0.0 | 0.97 |
| Global | 32.0 | 0.0041 | 44.0 | <0.0001 |

^a^LRT indicated lactation stage to be categorical in the mastitis model and linear in the culling model.

^b^ In previous 12 months prior to commencement of outbreak
